# Highly Adhesive Antibacterial Bioactive Composite Hydrogels With Controllable Flexibility and Swelling as Wound Dressing for Full-Thickness Skin Healing

**DOI:** 10.3389/fbioe.2021.785302

**Published:** 2021-12-23

**Authors:** Guanhua Lan, Suping Zhu, Dong Chen, Hua Zhang, Lijin Zou, Yuanlin Zeng

**Affiliations:** ^1^ Burn Center, The First Affiliated Hospital of Nanchang University, Nanchang, China; ^2^ Department of Pediatrics, Ningbo Yinzhou Second Hospital, Ningbo, China; ^3^ Department of Pathology, Ningbo Yinzhou Second Hospital, Ningbo, China; ^4^ Cixi Institute of Biomedical Engineering, Ningbo Institute of Materials Technology and Engineering, Chinese Academy of Sciences, Ningbo, China

**Keywords:** antibacterial, hydrogel, polyzwitterionic, gelatin, quaternized chitosan, controllable swelling, skin adhesiveness, wound healing

## Abstract

Polyzwitterionic hydrogels as skin wound dressings have been extensively studied owing to their superior antibacterial properties and skin adhesiveness, but their practical applications still suffer from a low adhesion strength and a high swelling ratio, which hinder the application of hydrogel for cutaneous healing. Here, we developed a novel biocompatible poly[2-(methacryloyloxy)ethyl]dimethyl-(3-sulfopropyl)ammonium hydroxide (PolySBMA) composite hydrogel with high stretchability, low swelling, strong skin adhesiveness, and antibacterial effect for enhancing wound healing. Naturally rigid polymers including quaternized chitosan methacrylate (QCSMA) and gelatin methacrylate (GelMA) are used as bioactive cross-linkers to endow PolySBMA/QCSMA/GelMA (SQG) hydrogel with a low swelling ratio and high bioactivity. The optimized hydrogel has excellent mechanical flexibility, with the ultimate tensile strength, tensile strain, modulus, and toughness of up to 344.5 kPa, 364%, 14.7 kPa, and 33.4 kJ m^−3^, respectively. The adhesiveness of the hydrogel to the skin tissue is as high as 38.2 kPa, which is critical for stopping the bleeding from the wound. The synergistic contributions from the PolySBMA and QCSMA endow hydrogel with good antibacterial properties against both Gram-positive *Staphylococcus aureus* and Gram-negative *Escherichia coli*. Moreover, the natural polymer cross-linked polyzwitterionic hydrogel shows good cell activity, hemocompatibility, and histocompatibility. The *in vivo* full-thickness skin defect model demonstrates that the SQG hydrogel efficiently improves the granulation tissue formation and collagen deposition. In summary, such superiorly skin-adhesive antibacterial biocompatible hydrogel with controllable flexibility and swelling holds great promise as wound dressings for acute wounds.

## Introduction

The human skin, as the external barrier of the body, faces a great risk of mechanical damage. Once the skin tissue is extensively damaged by various internal and/or external factors, the skin is hard to self-repair (Liang et al., 2019; Cui et al., 2020). To achieve large wound healing, a variety of bioactive wound dressings, including nanofibers, biocompatible membranes, porous foams, and hydrogels, have been developed to improve the formation of granulation tissue, revascularization, re-epithelialization, and remodeling (Clark et al., 2020; Tottoli et al., 2020). Among them, polymeric hydrogels have been extensively recognized for their soft/wet properties similar to extracellular matrices (ECMs), which make them promising for skin repair (Gupta et al., 2019). An ideal hydrogel not only should have the capability of self-adhesiveness that allows it as a physical barrier to stop the bleeding and avoid bacterial infections but also should provide multi-biofunctions to improve cell growth and granulation formation (Li et al., 2020; Op’t Veld et al., 2020; Tavakoli and Klar, 2020; Liang et al., 2021; Pei et al., 2021). Besides, having similar or superior mechanical properties to the skin and controllable swelling plays crucial roles in accelerating wound healing.

Zwitterionic polymers such as poly [2-(methacryloyloxy)ethyl]dimethyl-(3-sulfopropyl) ammonium hydroxide (PolySBMA) have widely been used as wound repair materials, attributing to their attractive biological and mechanical properties (Pei et al., 2020; Zhu et al., 2016; Wang J. et al., 2020; Li et al., 2021). For example, the PolySBMA can bond to the skin surface through the ion–dipole and dipole–dipole interactions between the zwitterionic groups of PolySBMA and the functional groups on the tissues. These strong interface interactions endow PolySBMA hydrogels with excellent adhesiveness to the skin. Moreover, these zwitterionic groups endow the hydrogels with effective antibacterial properties. However, owing to the high solvation of these zwitterionic groups, PolySBMA-based hydrogels mostly suffer from the limitation of large swelling and low bioactivity (Fang et al., 2020; Ye et al., 2016; Wang Z. et al., 2020). Excessive swelling easily leads to the fluid unbalance of the ECM *in vivo* (Ren et al., 2019). Furthermore, largely swollen hydrogel mismatches the wound shapes and even worsens the deformation of the local tissues, thus restricting their applications in tissue repair (Zhan et al., 2021). Besides, these hydrogels usually lack cell adhesion sites, resulting in slow cell migration and proliferation and thereby low skin tissue formation *in vivo*. Therefore, developing novel adhesive PolySBMA hydrogel with low swelling and high bioactivity to promote wound healing is highly desirable.

Natural polymers, such as hyaluronic acid, chitosan, and gelation, have excellent biocompatibility, biodegradability, and tissue affinity as compared with synthetic polymers (Zhu et al., 2019; Naomi et al., 2021). Introducing natural polymers into synthetic hydrogel networks would be helpful for their biological activity. Gelatin, as a polypeptide derived from collagen proteins, is a primary protein component of tissues (Alipal et al., 2021). It has been reported that polyzwitterionic hydrogels composited with gelatin facilitated the wound healing process by enhancing cell migration and proliferation, thereby promoting angiogenesis and neo-tissue formation (Lai et al., 2016). On the other hand, as one of the major cationic polymers, chitosan also promotes wound healing and has bacteriostatic effects and pH sensitivity due to the abundantly available amino groups (Bezrodnykh et al., 2021; Feng et al., 2021). For pH below its pKa value of 6.3, the free amino group (NH_2_) on the chitosan chains is protonated into the positively charged group (NH^3+^), making chitosan soluble in aqueous acid solutions (Choi et al., 2016). Oppositely, it is normally insoluble in physiological solutions including cell culture medium and acute skin wound microenvironment. Such characterization leads to the expulsion of water from chitosan-based hydrogels and eventually reduces the hydrogel volume. Therefore, it is promising for controlling network swelling by introducing chitosan into PolySBMA hydrogels.

The objective of this study was to fabricate strongly adhesive, bioactive hydrogels with antibacterial properties and low swelling for skin regeneration. The hydrogels are composed of zwitterionic [2-(methacryloyloxy)ethyl]dimethyl-(3-sulfopropyl) ammonium hydroxide (SBMA) and natural polymers including quaternized chitosan (QCS) methacrylate (QCSMA) and gelatin methacrylate (GelMA), denoted as the SQG hydrogel. Both QCSMA and GelMA act as biocompatible cross-linkers in the network. Moreover, GelMA is beneficial to improve the hydrogel’s bioactivity, and QCSMA tunes the anti-swelling and antibacterial properties (Xue et al., 2019). By adjusting component concentrations, a series of hydrogels with different rheology, morphology, mechanical properties, and adhesion and antibacterial properties were obtained and characterized. Furthermore, the biocompatibility of the SQG hydrogels was systematically investigated via fibroblast culture *in vitro* and subdermal implantation *in vivo*. Finally, the optimized SQG hydrogels were used as wound dressing for full-thickness skin wound repair in a mouse model to demonstrate their potential clinical uses. The histological changes related to tissue remodeling as well as collagen deposition were examined to evaluate the neo-tissue formation. All of these results demonstrated that the SQG hydrogel with excellent mechanical properties, self-adhesiveness, antimicrobial effects, and biocompatibility could be a suitable candidate for wound healing applications.

## Materials and Methods

### Chemicals

[2-(methacryloyloxy)ethyl]dimethyl-(3-sulfopropyl) ammonium hydroxide (SBMA, 97%), Gelatin (Type A), methacrylate anhydride (94%), and ammonium persulfate (APS) were obtained from Aladdin (Shanghai, China). Quaternary ammonium chitosan (QCS; degree of quaternary ≈80%) was purchased from Lushen Bioengineering (Nantong, China).

### Synthesis of Quaternary Ammonium Chitosan Methacrylate

Quaternary ammonium chitosan methacrylate (QCSMA) was prepared by the reaction of the free amino on QCS and methacrylate anhydride. First, QCS was dissolved in deionized water to 1% (wt/v) concentration. Methacrylate anhydride was then added to the solution with the molar ratio of anhydride to amino groups of 1. The reaction was carried out at 60°C for 3 h. The resulting solution was dialyzed in a dialysis bag with a cutoff molecular weight of 8,000–14,000 against deionized water for 5 days to remove the unreacted reagents, followed by lyophilization.

### Synthesis of Gelatin Methacrylate

The preparation process of GelMA was similar to that of the QCSMA reaction. Briefly, gelatin was dissolved in deionized water to 1% (wt/v) concentration. Methacrylate anhydride was then added to the above solution with the molar ratio of anhydride to amino groups of 1. The reaction was carried out at 60°C for 3 h. The resulting solution was dialyzed in a dialysis bag with a cutoff molecular weight of 8,000–14,000 against deionized water for 5 days to remove the unreacted reagents, followed by lyophilization. The chemical structures of GelMA were confirmed by ^1^H NMR on a Bruker (Billerica, MA, USA) NMR (400 MHz) with D_2_O as solvent.

### Preparation of SQG Hydrogels

The bioactive SQG hydrogels were prepared by a one-pot process. In a typical synthesis, an aqueous solution of GelMA [4% (wt/v)] was first prepared by vigorous stirring at 50°C. The QCSMA was further dissolved into GelMA solution to 4% concentration. After the addition of SBMA and initiator APS into the solution, the mixture was vigorously stirred for 10 min at 4°C and then degassed by centrifugation at 8,000 rpm. The formulations for the preparation are shown in Table 1. Subsequently, the mixed solution was transferred into a mold composed of a silicone rubber spacer (thickness = 1.5 mm) between two glass plates. The mold containing the precursor solution was wrapped in a sealed polyethylene plastic bag and then placed in a water bath at 60°C for 12 h to *in situ* initiate the copolymerization of SBMA, QCSMA, and GelMA. The resulting hydrogels were denoted as S_x_Q_y_G_z_, where S, Q, and G refer to SBMA, QCSMA, and GelMA with subscripts x, y, and z for their concentrations, respectively.

**TABLE 1:** Formulations of the precursor solutions for the preparation of S_x_Q_y_G_z_ hydrogels.

Hydrogel	SBMA (mol/L)	QCSMA (w/v%)	GelMA (w/v%)	APS (mol/L)
S_1_Q_4_G_4_	1	4	4	5 * 10^–3^
S_2_Q_4_G_4_	2	4	4	
S_3_Q_4_G_4_	3	4	4	
S_4_Q_4_G_4_	4	4	4	
S_4_Q_2_G_4_	4	2	4	
S_4_Q_6_G_4_	4	6	4	
S_4_Q_4_G_2_	4	4	2	
S_4_Q_4_G_6_	4	4	6	
S_4_Q_4_	4	4	0	
S_4_G_4_	4	0	4	

Note. SBMA, [2-(methacryloyloxy)ethyl]dimethyl-(3-sulfopropyl) ammonium hydroxide; QCSMA, quaternized chitosan methacrylate; GelMA, gelatin methacrylate; APS, ammonium persulfate.

### Characterization

#### Chemical Structures

The chemical structures of QCSMA and GelMA were analyzed by ^1^H NMR on a Bruker NMR (400 MHz) with D_2_O as solvent. The degree of methacrylate substitution (DS) of QCSMA was calculated as (%) = (%) = *A*_*H*_ (5.3 and 5.6)/*A*_*H*_ (3.1) × 80%, where AH (5.3 and 5.6) is the area of methylene protons peaks at 5.3 and 5.6 ppm, and the –N^+^(CH_3_)_3_ group protons peak at 3.1 ppm. The degree of substitution (*DS*) of GelMA was calculated as (%) = 1 − (plysine methylene proton of GelMA)/(plysine methylene proton of Gelatin) × 100%. The QCSMA was further characterized using a Fourier transform infrared (FTIR) spectrometer (Micro-FTIR, Agilent Cary 660, Santa Clara, CA, USA) in a transition mode. All the spectra were obtained with 32 scans and a resolution of 4 cm^−1^ in the range of 4,000–400 cm^−1^.

#### Mechanical and Rheological Tests

The mechanical performances of hydrogels were investigated using a Universal Testing Machine (Instron 5567, Norwood, MA, USA). Hydrogel samples were cut into dumbbell-shaped specimens (2 mm width × 10 mm high × 1.5 mm thickness) for the tensile test. The crosshead speed was set to 100 mm min^−1^. The fracture toughness of each sample was calculated by integrating the area under the stress–strain curve. Rheological testing was carried out on a TA Instrument (New Castle, DE, USA) DHR-2 Rheometer, using a 20-mm parallel plate with a gap of 1,500 μm. Temperature sweeps were performed from 5°C to 40°C and then back to 5°C at 10 rad s^−1^ of angular frequency by using 1% constant strain. Frequency sweeps were performed from 0.1 to 100 rad s^−1^ by using 1% strain. The periphery was sealed by silicone oil to prevent water evaporation.

#### Equilibrium Swelling Ratio of SQG Hydrogels

The equilibrium swelling ratio (ESR) and stability of the hydrogels were analyzed through a swelling test. The fresh hydrogels were then immersed into 25 ml of phosphate-buffered saline (PBS) (pH = 7.4) at 37°C. The weights of all hydrogels were recorded at regular intervals until they were kept constant (*W*_*s*_). Subsequently, the samples were completely dried under vacuum, and the dry weights were measured (*W*_*d*_). ESR was calculated as (%) = (*W*_*s*_ − *W*_*d*_)/*W*_*d*_ × 100.

#### Adhesion Strength Measurements of the Hydrogel

The lap shear method was carried out to evaluate the adhesion performances of hydrogels. Typically, a hydrogel was sandwiched between two pieces of glass with a contact area of 25 × 25 mm^2^. Subsequently, the sandwich-like specimen was pressed by applying a force of 1 N for 5 min to achieve sufficient contacting. The two ends of the assembly were fixed on a Universal Testing Machine (Instron 5567, USA) and pulled at a shear rate of 100 mm min^−1^ until the breaking point. The adhesion strength was determined by dividing the maximum force by the adhesive contact area. Hydrogels were sandwiched between two pieces of rubber, Teflon, and porcine skin to measure the adhesion performance of the S_4_Q_4_G_4_ hydrogel with them. All tests were employed more than 3 times, and the results were shown as mean SD.

#### Antibacterial Activity Evaluation

The antibacterial activity of hydrogel was evaluated by a direct coincubation method using *Escherichia coli* (ATCC 8739) and *Staphylococcus aureus* (ATCC 29213) according to the reference (Zhao et al., 2015). Briefly, the S_4_Q_4_G_4_ hydrogels (8 mm diameter × 1.5 mm height) were separately placed in a 48-well plate. Bacterial suspension measuring 5 μl with 10^5^ CFU·ml^−1^ was spread on the surface of hydrogels, and the same suspension was added in 1.0 ml of PBS as the control. After incubation at 37°C in a relatively humidified atmosphere for 6 h, 1 ml of sterilized PBS was added to each well to resuspend any bacterial survivors. The resuspension measuring 10 μl was further seeded on the appropriate agar plate. After incubation for 18 h at 37°C, the colony-forming units (CFU) on the agar plate were counted. Tests were performed in triplicate (n = 3) for each group, and the killing ratio (%) was calculated by the following equation:

$$kill\text{\%}=\frac{C_{contr}\;-C_{hydr}\;}{C_{contr}}\times 100\%$$

where *C*_*contr*_ and *C*_*hydr*_ were the cell count of the control survivor and cell count on hydrogels, respectively.

#### Biocompatibility Evaluation

For cytocompatibility studies, the NIH 3T3 cells supplied by the medical school of Ningbo University were seeded onto the sterilized SQG hydrogels with a density of 1 × 10^6^ ml^−1^. The SQG hydrogels were immersed in growth media [low-glucose Dulbecco’s Modified Eagle Medium (DMEM; Gibco, Grand Island, NY, USA) supplemented with 10% fetal bovine serum (FBS, Gibco) and 1% penicillin/streptomycin (Gibco)]. After 1 and 2-day culture in a humidified incubator at 37°C with 5% CO_2_, cells were stained with phalloidin (Cytoskeleton, Inc., Denver, CO, USA) and 4′,6-diamidino-2-phenylindole (DAPI; Beyotime Institute of Biotechnology, Inc., Jiangsu, China) according to the protocol (Beyotime, China). Images were visualized using a Confocal Laser Scanning Microscopy (CLSM; Leica TCS-SP8, Wetzlar, Germany). The cell proliferation on the hydrogels was also assessed by using the Cell Counting Kit-8 (CCK-8) assay according to the protocol.

The histocompatibility of hydrogels *in vivo* was evaluated by subdermal implantation as the reference described (Teng et al., 2021; Zhou et al., 2020). Eight-week-old Institute of Cancer Research (ICR) mice (25–30 g) were housed in the Animal Service Centre of Ningbo University. All surgical procedures were approved by the Institutional Animal Ethical Committee (IAEC) of Ningbo University, Ningbo, China. Briefly, the mice were anesthetized by isoflurane and fixed on a surgical corkboard. The back hair was shaved and sterilized with betadine. Subsequently, one incision about 8 mm in length was created by bistoury. The S_4_Q_4_G_4_ hydrogels (8 mm diameter × 1.5 mm height) were placed in the subcutaneous cavity. Tissue samples connected with hydrogel were harvested at 7 and 14 days, immediately fixed in 10% neutral buffered formalin, and embedded in paraffin. Samples were fixed with 4% paraformaldehyde overnight at 4°C, then embedded in paraffin, and cross-sectioned to 6-μm-thick slices. The sections were immunohistochemically stained with CD 68 and observed using a Confocal Laser Scanning Microscopy (CLSM) (Leica TCS-SP8, Germany).

Red blood cells from rabbits were used to evaluate the blood compatibility of the SQG hydrogel according to the previously reported protocol. First, the optical density of the rabbit whole blood diluted 0.9% NaCl solution was regulated to 0.8 ± 0.3 at 545 nm in distilled water. Subsequently, hydrogel samples (8 mm diameter × 1.5 mm height) were incubated in the 0.9% NaCl solution-diluted blood at 37°C for 30 min. Deionized water-diluted blood and 0.9% NaCl solution-diluted blood were used as positive control and negative control, respectively. After incubation, the blood was centrifugated at 1,000 rpm for 10 min to collect the supernatants, and then the absorbance was measured at 545 nm. Hemolysis ratio was calculated as (%) = (*A*_*h*_ − *A*_*n*_)/(*A*_*p*_ − *A*_*n*_) × 100%, where *A*_*h*_, *A*_*n*_, and *A*_*p*_ represent the absorbance of the blood supernatants incubated with the SQG hydrogel, the negative control, and the positive control, respectively.

A skin irritation test was performed according to the reported method (Fang et al., 2020; Ye et al.). The back hair of the mice anesthetized by isoflurane was shaved and sterilized with betadine. The area is divided into two equal parts, where the S_4_Q_4_G_4_ hydrogel (8 mm in diameter and 1.5 mm in thickness) and medical gauze wetted with PBS as control were placed. The samples were fixed using a Transparent Film Dressing (3M Health Care, Saint Paul, MN, USA). After being treated for 24 h, the contact sites were observed and recorded.

#### Acute Cutaneous Wound Repair

The *in vivo* wound healing experiments were carried out by a full-thickness skin defect model. ICR mice (25–30 g, 8 weeks of age) were employed and divided into control and hydrogel groups randomly. Each group contained 10 mice. Immediately prior to surgery, mice were anesthetized by isoflurane and fixed on a surgical corkboard. The back of the mice was shaved and sterilized with betadine. Two incisions about 8 mm diameters were created by needle biopsy. For the control group, the wounds were protected by using Transparent Film Dressing Frame Style (3M Health Care, USA). For the hydrogel groups, the S_4_Q_4_G_4_ hydrogels were applied to the wounds. The wound diameter was measured on the 3rd, seventh, 10th, 12th, 14th, 18th, and 21st days. Wound contraction (%) was calculated by the following formula:

$$\text{Wound}\;\text{contraction}\;\text{\% }=\;\left({\text{Area}}_{\left(\text{0 day}\right)}-{\text{Area}}_{\left(\text{n day}\right)}\right)/\left({\text{Area }}_{\left(\text{0 day}\right)}\right)\text{×}100\text{\%}$$

where “n” represents the monitored day.

For evaluation of skin regeneration in the wound area, samples collected on the seventh, 14th, and 21st days were fixed with 4% paraformaldehyde overnight at 4°C, then embedded in paraffin, and cross-sectioned to 6-μm-thick slices. The sections were stained with H&E and Masson’s trichrome, followed by subsequent stereological analysis. To assess the deposition of collagen fibrils in the wounds, tissue sections were stained with Picrosirius red dye and scanned under a polarizing microscope (X51, Olympus, Tokyo, Japan). ImageJ image analysis software was used to measure the wound area, neovascularization number, granulation tissue thickness, and collagen-occupied regions.

#### Statistical Analysis

The experimental data were analyzed by Student’s t-tests for comparison between two groups or the ANOVA (Tukey’s post-hoc test) for multiple comparisons. All results were presented as mean ± SD. *p* < 0.05 was considered to be statistically significant.

## Results and Discussion

### Preparation of SQG Hydrogels

Considering the desired tissue adhesiveness, antibacterial properties, and biocompatibility in practical application, naturally derived chitosan, gelatin, and zwitterionic [2-(methacryloyloxy)ethyl]dimethyl-(3-sulfopropyl)ammonium hydroxide (SBMA) were carefully selected to prepare hydrogels *via* covalent interactions. In chitosan derivations, QCS modified by glycidyltrimethylammonium chloride not only endows chitosan with a good water solubility but also provides excellent inherent antibacterial properties for the hydrogels due to the existence of quaternary ammonium cation. In order to endow QCS with the capacity of copolymerization by free radical polymerization, it was further modified with methacrylate anhydride to obtain the double bonds (Supplementary Figure S1A). Gelatin possesses good biocompatibility, biodegradability, and easily modified chemical properties, which make it one of the most competitive components of skin repair materials. Gelatin was also modified with double bonds using methacrylate anhydride (Supplementary Figure S1B). ^1^H NMR demonstrated that the substitution degree of QCSMA and GelMA is 6% and 43%, respectively (Supplementary Figure S1B; Supplementary Figure S2).

**SCHEME 1:**
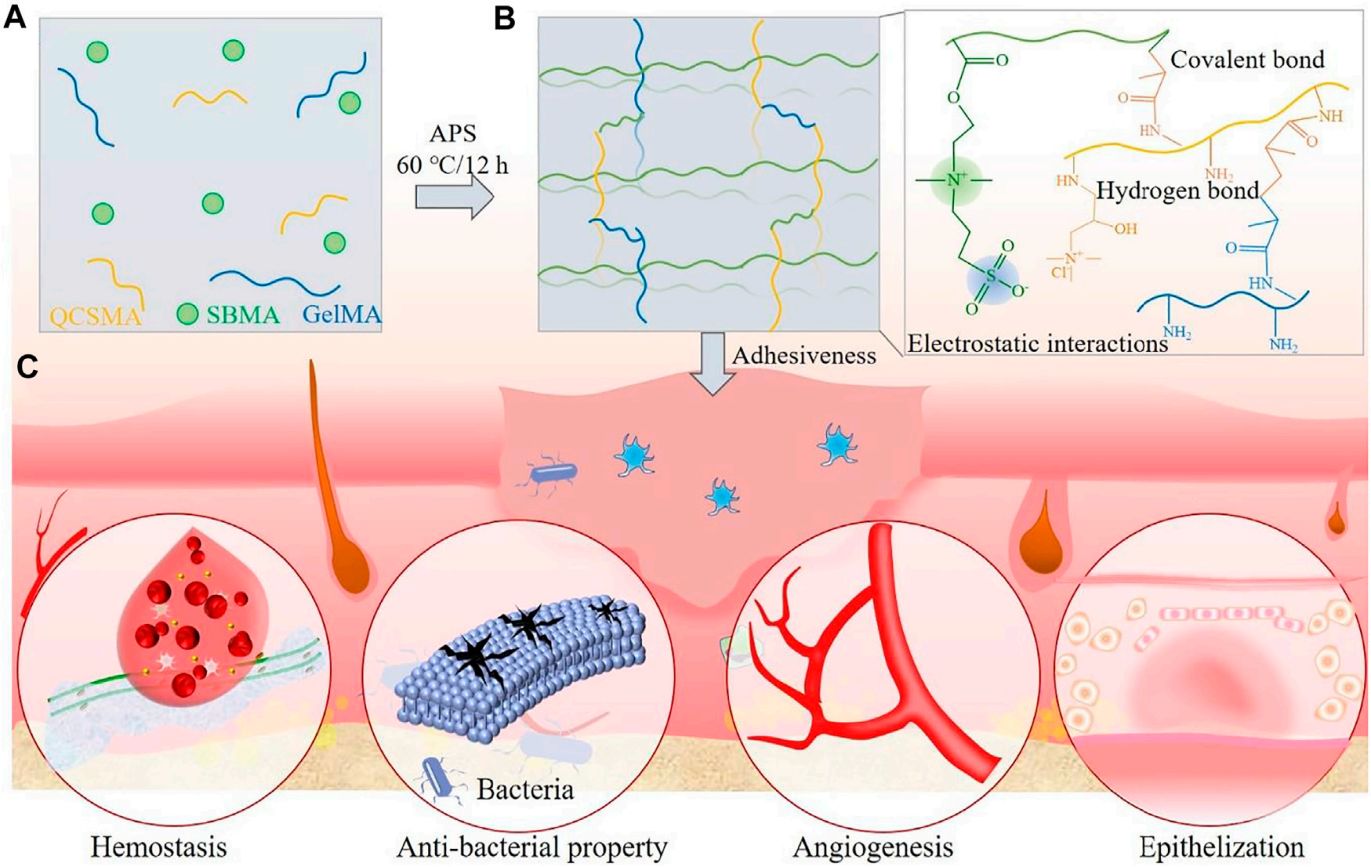
Schematic illustration of the PolySBMA/QCSMA/GelMA (SQG) hydrogel **(A)** with strong chemical bonding and dynamical physical interactions **(B)** for wound repair **(C)**. PolySBMA, poly(sulfobetaine methacrylate); QCSMA, quaternized chitosan methacrylate; GelMA, gelatin methacrylate.

The synthesis procedures of PolySBMA/QCSMA/GelMA (SQG) hydrogel and further applications in cutaneous wound repair are depicted in Scheme 1. The hydrogels were obtained by free radical polymerization of SBMA, QCSMA, and GelMA with the APS initiator system (Scheme 1A). In the hydrogel, the PolySBMA chains are chemically cross-linked by QCSMA and GelMA (Scheme 1B). The sulfonic acid anions of PolySBMA also interact with ammonium cations of QCSMA and GelMA *via* electrostatic force to form additional electrostatic interactions. Besides, the abundant hydroxyl and carboxylic acid groups of GelMA are involved in the hydrogen bonding with PolySBMA and QCSMA units (Liang et al., 2020). Therefore, the SQG hydrogel has a synergetic dual cross-linking structure in a 3D network including strong chemical cross-linking bonding and dynamical physical interactions. The chemical cross-linking in the hydrogel could stabilize the deformation, while the physical interactions are beneficial to release energy during deformation and endow hydrogel with reverse adhesion properties. Benefiting from the synergetic network, the SQG hydrogel would have controllable mechanical and swelling properties, tissue adhesiveness for hemostasis, antibacterial effect for killing bacteria, and bioactivity for angiogenesis and epithelization, showing great potential for skin wound repair (Scheme 1C).

### Mechanical Properties and Viscoelasticity of SQG Hydrogel

**FIGURE 1:**
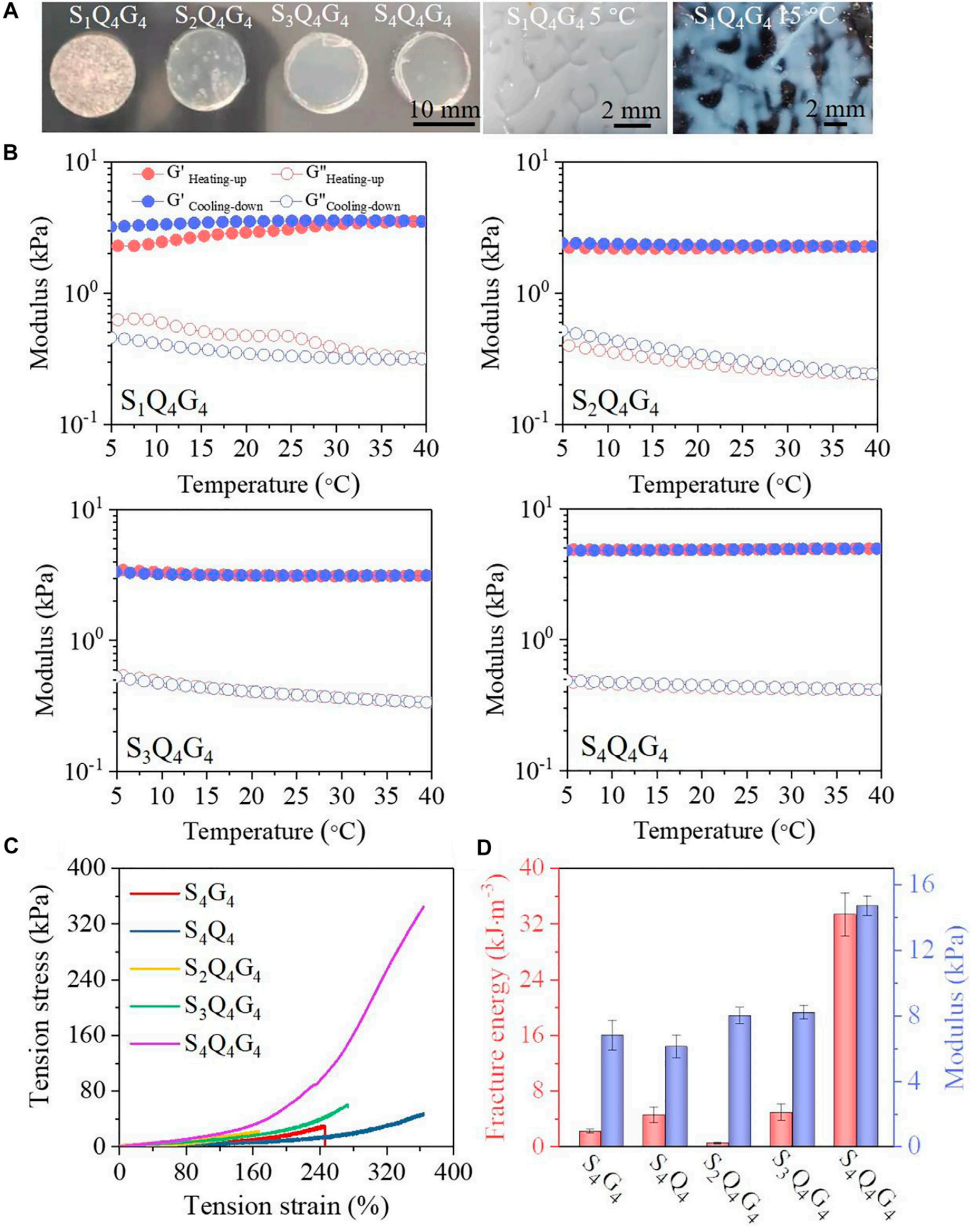
**(A)** Photographs of S_1_Q_4_G_4_, S_2_Q_4_G_4_, S_3_Q_4_G_4_, and S_4_Q_4_G_4_ hydrogels at room temperature and S_1_Q_4_G_4_ at 5°C and 15°C. **(B)** Temperature dependency of storage modulus (G′) and loss modulus (G″) of S_1_Q_4_G_4_, S_2_Q_4_G_4_, S_3_Q_4_G_4_, and S_4_Q_4_G_4_ hydrogels. **(C,D)** Tensile stress–strain curves **(C)** and the modulus at a strain of 20% and fracture energy **(D)** of S_4_Q_4_, S_4_G_4_, S_1_Q_4_G_4_, S_2_Q_4_G_4_, S_3_Q_4_G_4_, and S_4_Q_4_G_4_ hydrogels.

To optimize the polymer blend for use as a strongly adhesive hydrogel with desired mechanical property, the SQG with different QCSMA [2%–6% (wt/v)] and GelMA [2–6% (wt/v)] concentrations are first investigated when the SBMA concentration was fixed at 4 mol L^−1^. The SQG hydrogels exhibited enhanced tensile performances as compared with the PolySBMA hydrogels only with QCSMA or GelMA chains (Supplementary Video S1). Moreover, the tensile property of the SQG hydrogels first increases with the QCSMA or GelMA concentration increasing from 2% to 4% and then decreases when reaching 6%. Therefore, the experimentally proposed QCSMA and GelMA concentrations were both fixed at 4% in the networks. Furthermore, the effect of SBMA concentrations of 1, 2, 3, and 4 mol L^−1^ on hydrogel’s properties was symmetrically investigated. The hydrogels at 5°C are shown in Figure 1A. It is obviously observed that the S_1_Q_4_G_4_ hydrogel at 5°C is opaque. With the SBMA concentration increasing from 1 to 4 mol L^−1^, the optical transmittance of the SQG hydrogels significantly increases. This is attributed to the decrease of inherent hydrogen bonding between GelMA chains with the increase of SBMA concentrations. Analysis of the storage (G′) and loss (G″) moduli of the four hydrogel compositions demonstrated that the SBMA concentrations directly influenced the hydrogel temperature-sensitive properties when varying the cyclic temperature from 5°C to 40°C (Figure 1B). Especially, the G′ and G″ of the S_1_Q_4_G_4_ hydrogel significantly separate during the heating and cooling processes, while the moduli of the S_2_Q_4_G_4_, S_3_Q_4_G_4_, and S_4_Q_4_G_4_ hydrogels almost coincided. Moreover, the G″ of the S_1_Q_4_G_4_ and S_2_Q_4_G_4_ hydrogels obviously decreases with the temperature increase, but a little G″ variation is observed in the S_3_Q_4_G_4_ and S_4_Q_4_G_4_ hydrogels. These results confirm that more hydrogen bonding formed between GelMA chains in a lower PolySBMA network, resulting in a greater hysteresis degree of moduli with cyclic temperature.

The influences of SBMA concentration on the hydrogel mechanical properties were analyzed in terms of rheological properties and tensile experiments. It was found that the G′ varied a little when varying the angular frequency from 1 to 100 rad s^−1^, indicating predominantly elastic SQG hydrogels (Supplementary Figure S3). For the S_1_Q_4_G_4_, S_2_Q_4_G_4_, S_3_Q_4_G_4_, and S_4_Q_4_G_4_ hydrogels, the G′ values at 10 rad s^−1^ are approximately 1.8, 2.3, 3.9, and 4.6 kPa, respectively. The maximum loss modulus at 100 rad s^−1^ was in the order of 1.5 kPa for the S_4_Q_4_G_4_ hydrogel, indicating that more energy is dissipated in the hydrogels than is stored, which could effectively toughen the S_4_Q_4_G_4_ hydrogel during large tension to release energy. Because the S_1_Q_4_G_4_ hydrogel was so brittle that it was difficult to tension, the S_2_Q_4_G_4_, S_3_Q_4_G_4_, and S_4_Q_4_G_4_ hydrogels were further assessed by tensile tests. The mechanical performances of SQG presented a positive correlation with SBMA concentration, with a tensile strength of approximately 20.2 kPa at 165% strain, 60.7 kPa at 273% strain, and 344.5 kPa at 364% strain for the S_2_Q_4_G_4_, S_3_Q_4_G_4_, and S_4_Q_4_G_4_ hydrogels, respectively (Figure 1C). These results demonstrate an intense PolySBMA concentration-dependent effect on the mechanical properties of the SQG hydrogel. Additionally, the fracture tensile strength of the S_4_Q_4_G_4_ hydrogel is higher than that of S_4_Q_4_ (30.1 kPa at a strain of 243%) and S_4_G_4_ hydrogels (44.7 kPa at a strain of 360%), while that of the S_2_Q_4_G_4_ and S_3_Q_4_G_4_ is lower, further suggesting the positive correlation of SBMA concentration to hydrogels’ mechanical properties.

The tensile modulus was calculated from stress–strain curves, which also shows a positive correlation with SBMA concentration with values of 8.0, 8.2, and 14.7 kPa for the SQG hydrogels with 2, 3, and 4 mol L^−1^ SBMA, respectively (Figure 1D). The corresponding fracture energy calculated by integrating the area under the stress–strain curve increases from 0.5 to 4.9 and 33.4 kJ mol^−3^. The moduli and fracture energy of S_4_G_4_ and S_4_Q_4_ hydrogels, respectively, were 6.8 kPa and 2.2 kJ mol^−3^, and 6.1 kPa and 4.6 kJ mol^−3^. These results are much less than those of the S_4_Q_4_G_4_ hydrogel. The mechanical reinforcement results from the synergetic dual cross-linking structure in a 3D network (Scheme 1B). The increase of covalent cross-linking with the increase of SBMA concentration improves the stiffness. Besides, extensive additional hydrogen bonding and electrostatic interactions in the network were formed between PolySBMA, QCSMA, and GelMA chains to dissipate energy upon external loading. Benefiting from the synergistic effect, the S_4_Q_4_G_4_ hydrogel displayed excellent flexibility and adhesiveness during elongation and relaxation, bending, and twisting (Supplementary Video S2). After the external force was removed, these deformed hydrogels could recover to their original states. Therefore, the formation from the covalent bonding and intra/intermolecular interactions results in a flexible SQG hydrogel.

### Swelling Properties of SQG Hydrogel

**FIGURE 2:**
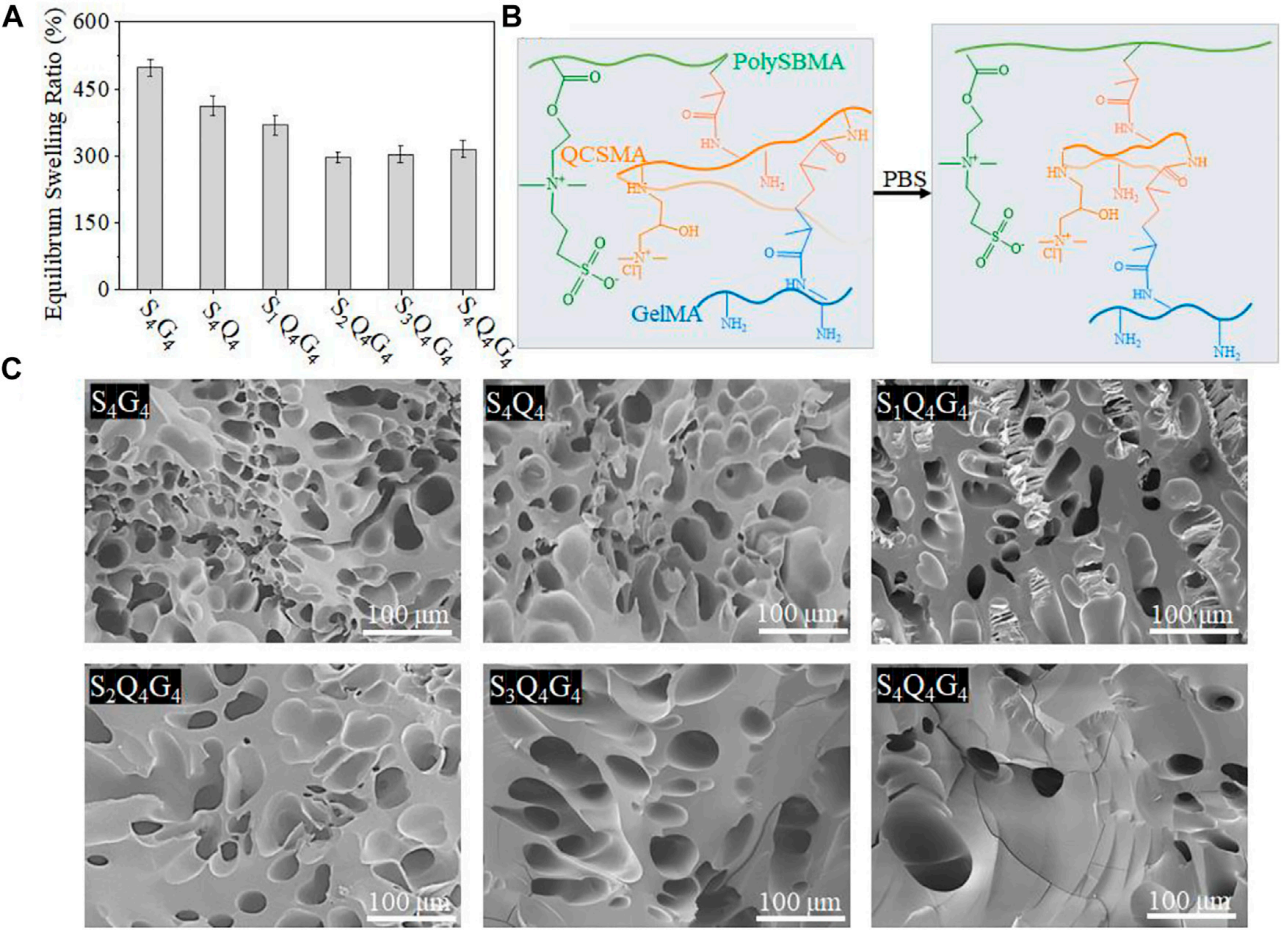
**(A)** Equilibrium swelling ratio of S_4_Q_4_, S_4_G_4_, S_1_Q_4_G_4_, S_2_Q_4_G_4_, S_3_Q_4_G_4_, and S_4_Q_4_G_4_ hydrogels in PBS (pH 7.4) at 37°C. **(B)** The schematic illustration of swelling for hydrogel network. **(C)** Cross-sectional SEM images of freeze-dried S_4_Q_4_, S_4_G_4_, S_1_Q_4_G_4_, S_2_Q_4_G_4_, S_3_Q_4_G_4_, and S_4_Q_4_G_4_ hydrogels. PBS, phosphate-buffered saline; SEM, scanning electron microscopy.

The SQG hydrogel as a potential wound dressing is required to controllably swell to absorb effusion of tissue fluid but avoid excessive absorption of normal tissue fluid, which would be negative to the wound healing. The swelling behavior of the SQG hydrogels within PBS (pH 7.4) was evaluated. PBS was used here to simulate the fluid phase present in the native skin tissue. After 24 h, the degree of swelling reached 185.4%, 165.3%, 142.1%, 108.5%, 114.6%, and 120.1% for S_4_Q_4_, S_4_G_4_, S_1_Q_4_G_4_, S_2_Q_4_G_4_, S_3_Q_4_G_4_, and S_4_Q_4_G_4_, respectively (Figure 2A). Consequently, the swelling of hydrogels was significantly suppressed by introducing QCSMA and GelMA into any SBMA concentrations from 1 to 4 mol L^−1^. The SQG hydrogels showed lower swelling degrees than those of the S_4_Q_4_ and S_4_G_4_ hydrogels, indicating a coordinate repression through the SQG cross-linking density to counter the osmotic pressure of additional water molecules. Therefore, although increasing PolySBMA concentration preferably binds a greater degree of water molecules through the solvation of zwitterionic groups that result in a larger amount of water being contained in the hydrogel networks, the cantic QCSMA and GelMA could endow the hydrogels with a high level of stability via intra/intermolecular interactions (Figure 2B). In line with this, scanning electron microscopy (SEM) images of the freeze-dried S_1_Q_4_G_4_, S_2_Q_4_G_4_, S_3_Q_4_G_4_, and S_4_Q_4_G_4_ hydrogels potentially suggest that the hydrogels became progressively less porous and much denser than S_4_G_4_ and S_4_Q_4_ hydrogels when increasing the SBMA concentration from 1 to 4 mol L^−1^ (Figure 2C).

### Adhesion Property of Hydrogel

**FIGURE 3:**
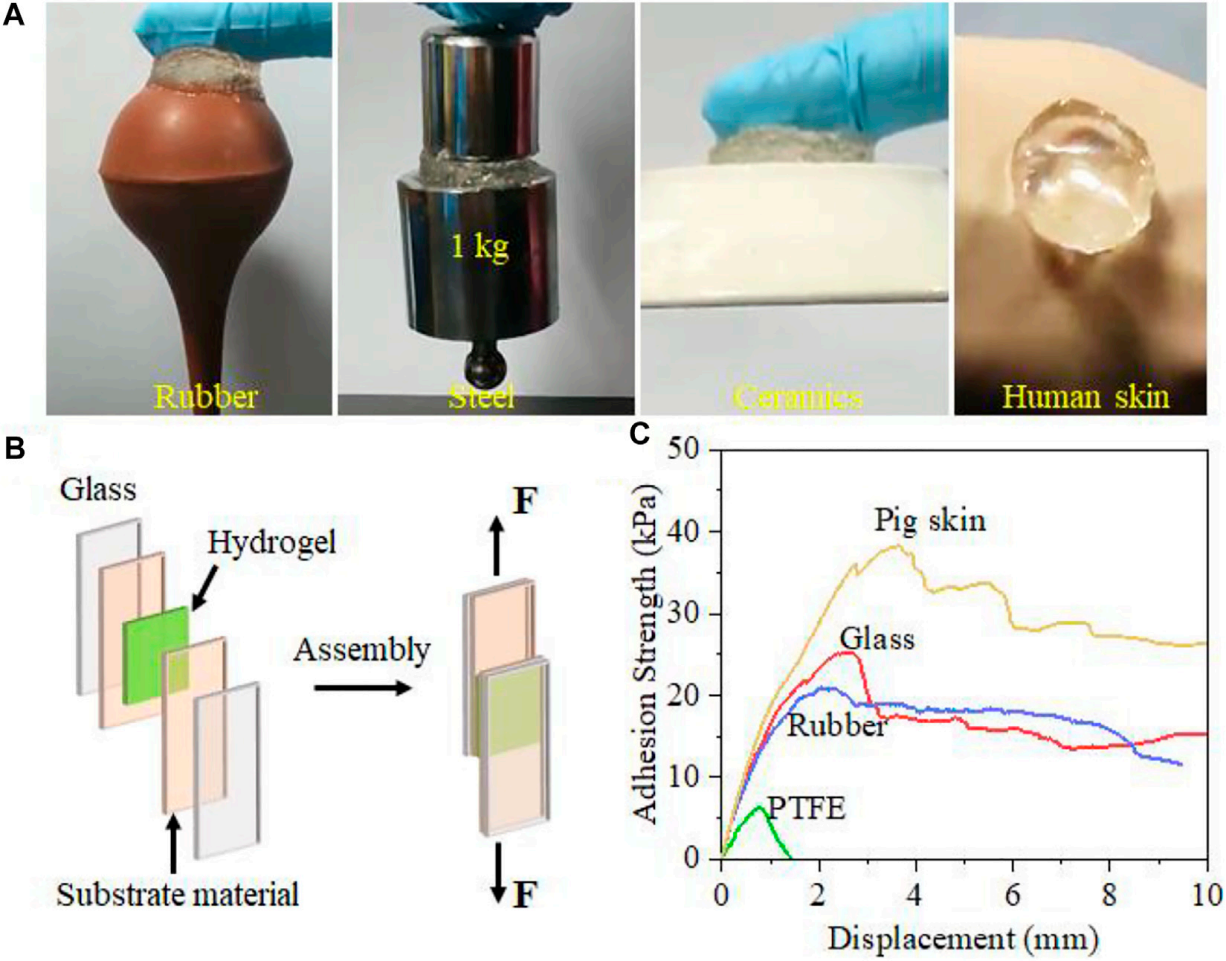
Adhesion of S_4_Q_4_G_4_ hydrogel to diverse substrates. **(A)** Photos of the universally self-adhesive S_4_Q_4_G_4_ hydrogel attached on rubber, steel weight, ceramics, and human skin. **(B)** Schematic illustration of the lap shear test. **(C)** Representative adhesion strength–displacement curves of lap shear tests on polytetrafluoroethylene (PTFE), rubber, glass, and pigskin glued by the hydrogel. Biocompatibility and antibacterial Effect of SQG hydrogel.

Excellent adhesion performance is also necessary for promoting the wound repair process. The zwitterionic PolySBMA and cationic QCSMA units endow the SQG hydrogel with a universal self-adhesive property *via* noncovalent interactions. As shown in Figure 3A, the hydrogel can firmly adhere to diverse surfaces including rubber, stainless steel, ceramics, and the human skin. Essentially, the adhesion performance is associated with the strong hydrogen bonding, ion–dipole, and dipole–dipole interactions with the substrate on the interface. Therefore, the SBMA content plays a critical role in the adhesion strength of hydrogel on the substrate. Through hanging a load of 1.0 kg by the hydrogel, it is found that the adhesion performance increases with the zwitterionic monomer content in the hydrogels (Supplementary Video S3). When the SBMA concentration is 2 mol L^−1^, the adhesiveness of the hydrogel is low. The S_2_Q_4_G_4_ hydrogel failed to hang the load up. As the SBMA content increases to 3 mol L^−1^, the adhesion ability of the hydrogel partly increases, but the adhesion failure for raising the load still happened. As the content of SBMA was raised to 4 mol L^−1^, the load was successfully adhered by the hydrogel and vertically hung in the air. After that, the hydrogel can be easily and completely peeled off without any residue, which brings convenience and comfortability for dressing use. Additionally, an assembly consisting of rubber and the 1.0-kg load adhered by the hydrogel could withstand a load of 1 kg after soaking in water (Supplementary Video S4). This phenomenon demonstrates excellent water-proof adhesion, which is critical for adhesion on wet bio-tissues. When adhering hydrogel to the dorsal skin of the mice, the S_4_Q_4_G_4_ hydrogel could tightly attach to the tissue and resist an intense movement as compared with the traditional medical gauze, suggesting a potential application even during body movements (Supplementary Video S5, Supplementary Video S6). The above excellent adhesiveness for dry, wet, and moving surfaces is principally attributed to abundant dipole moments from the high SBMA content, resulting in strong ion–dipole and dipole–dipole interactions at the contact interface. Besides, QCSMA and GelMA are capable of binding to many surfaces through hydrogen bonding, electrostatic interactions, and Schiff-based reaction.

The adhesion strength of hydrogel to diverse materials was evaluated by using lap shear tests (Figure 3B). Various assemblies consisting of the S_4_Q_4_G_4_ hydrogel sandwiched between a pair of glass slides, pigskins, and rubber or polytetrafluoroethylene (PTFE) (adhesion area: 25 × 25 mm^2^) were stretched from both ends until separation (adhesion failure) or fracture (cohesion failure). Figure 3C shows representative adhesion strength–displacement curves for the S_2_Q_4_G_4_ hydrogels on glass, rubber, PTFE, and pigskin. The maximum strength is taken as the interface adhesion strength for the interface failure. The adhesion strengths of the S_4_Q_4_G_4_ hydrogel for pigskin, glass, rubber, and PTFE, respectively, were 38.2, 25.4, 20.1, and 6.6 kPa. Remarkably, the adhesion strength of the S_4_Q_4_G_4_ hydrogels on pigskin (38.2 kPa) was far higher than that of fibrin glue (16.5 kPa). These results indicate the great potential of the hydrogels to form robust adhesion to the skin *in vivo*.

**FIGURE 4:**
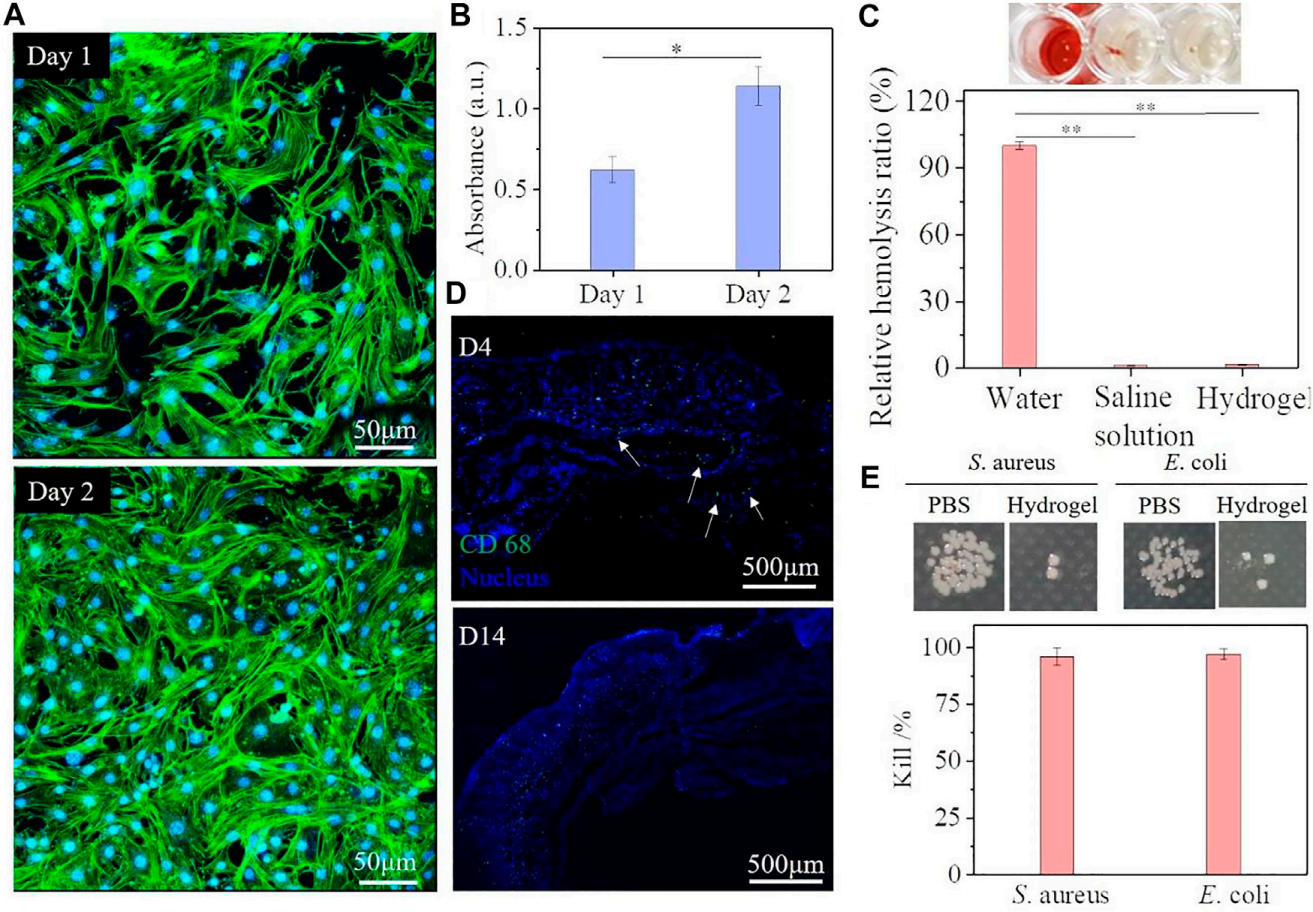
Biocompatibility and antibacterial effect evaluation of the S_4_Q_4_G_4_ hydrogel. **(A,B)** Fluorescent staining **(A)** and CCK-8 assay **(B)** of 3T3 cells seeded on the S_4_Q_4_G_4_ hydrogel for 1 and 2 days. **(C)** Fluorescent immunohistochemical staining with CD68 (green) and DAPI (blue) for macrophages after 4 and 14 days of subcutaneous implantation. **(D)** Hemolysis ratio of the S_4_Q_4_G_4_ hydrogel, deionized water, and saline solution. **(E)** Antibacterial activity against *Staphylococcus aureus* and *Escherichia coli* of S_4_Q_4_G_4_ hydrogels. CCK-8, Cell Counting Kit-8.

Good biocompatibility is an essential factor for hydrogel dressing. To assess the cytocompatibility of the SQG hydrogels *in vivo*, the 3T3 fibroblast cells of 1 × 10^6^ cells·ml^−1^ were seeded on the SQG hydrogels with different concentrations of SBMA for 24 h. The adhesion and spread morphologies of fibroblasts on the SQG hydrogels were imaged by using a CLSM (Figure 4A, Supplementary Figure S4). Typically, these fibroblasts uniformly adhered to the surface of the S_4_Q_4_G_4_ hydrogels. They maintained a similar morphology and formed a compact cell–cell interaction after incubation of 2 days, indicating that all the SQG hydrogels are cytocompatible scaffolds for fibroblast adhesion and spread. Furthermore, the CCK-8 assay was further used to quantitatively evaluate the proliferation of fibroblasts on days 1 and 2 (Figure 4B). The optical density values at 450 nm of the samples showed an increase over incubation time, indicating an increase in cell proliferation. These results consistently demonstrate the excellent cytocompatibility of the SQG hydrogel.

Hemocompatibility and histocompatibility *in vivo* as important indexes are fundamental for successful wound dressing applications. Many reported cytocompatible polyzwitterionic and cationic polymers suffer from significant hemolytic side effects that hamper their clinical application. Therefore, the hemocompatibility of the S_4_Q_4_G_4_ hydrogel was evaluated through a hemolysis activity assay. As shown in Figure 4C, the S_4_Q_4_G_4_ hydrogel remained nonhemolytic and almost indistinguishable from that of the negative control group (0.9% NaCl solution), while the deionized water group was bright red. The quantitative data show that the S_4_Q_4_G_4_ hydrogel exhibits a very low hemolysis ratio (1.4%) that was near to 1.1% of 0.9% NaCl solution, indicating the best hemocompatibility. Additionally, the S_4_Q_4_G_4_ hydrogel was implanted subcutaneously into ICR mice to evaluate *in vivo* inflammatory response. Hydrogel-surrounded skin tissues after 4 and 14 days were assessed by fluorescent immunohistochemical staining with CD68 for macrophages. As shown in Figure 4D, CD68^+^ macrophage invasion (green fluorescence) at the hydrogel–tissue interface is observed at day 4 but almost disappears at day 14, indicating a mild and early inflammatory response to the hydrogel. These results further imply that the SQG hydrogel possesses excellent biocompatibility, making them potentially promising for skin repair.

Bacterial infection has been considered one of the most challenging issues in wound treatment. Thus, the self-adhesive SQG hydrogel with antibacterial ability is of great value during clinical applications. *S. aureus* (Gram-positive bacteria) and *E. coli* (Gram-negative bacteria) were chosen as the representative bacteria. Higher antibacterial effect of the S_4_Q_4_G_4_ hydrogel was observed compared with the group from PBS solution, suggesting a broad-spectrum antibacterial property against Gram-positive *S. aureus* and Gram-negative *E. coli* (Figure 4E). After being in contact with hydrogels for 6 h at 37°C, more than 96% of *S. aureus* and 97% of *E. coli* were killed by the S_4_Q_4_G_4_ hydrogel. Such superior antibacterial capability is mainly attributed to the abundant zwitterionic groups and cationic charges produced by homogeneous quaternization that could efficiently disrupt the cell member after incubation (Qu et al., 2018).

#### *In Vivo* Skin Wound Healing in a Full-Thickness Skin Defect Model

**FIGURE 5:**
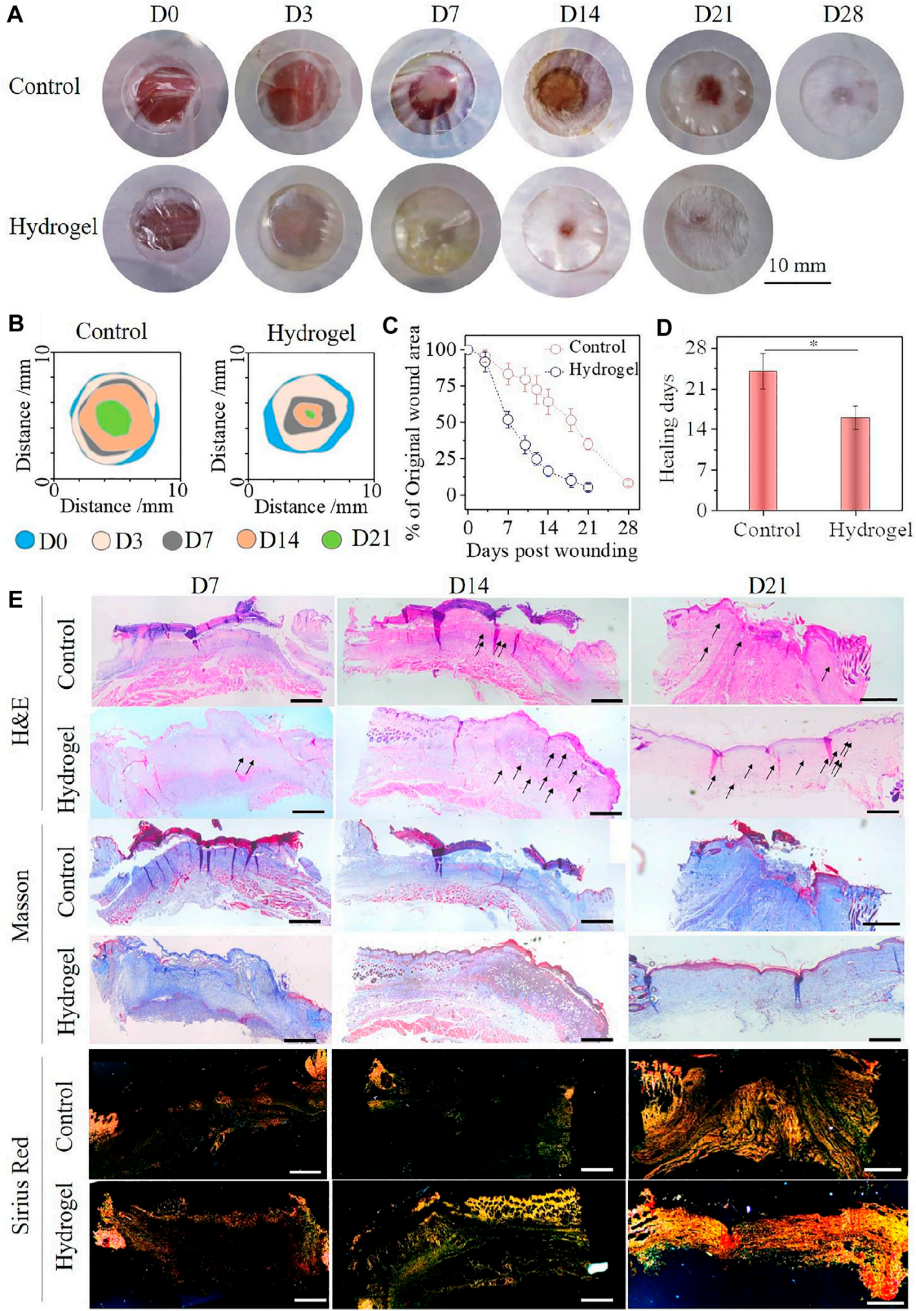
**(A)** Representative photographs of the acute skin wounds after being treated with S_4_Q_4_G_4_ hydrogel group and Tegaderm film control groups at each time point. **(B)** Traces of wound-bed closure during 21 days for each treatment. **(C)** Wound closure curves with hydrogel and control treatments. **(D)** Average healing times for each treatment. **(E)** H&E, Masson’s trichrome stains, and Picrosirius red staining of wound tissues. Scale bar: 200 μm.

Such excellent overall performance made the SQG hydrogel more applicable as a wound dressing for enhancing skin healing. To evaluate the repair efficacy of the hydrogel dressing *in vivo*, a full-thickness excisional wound model with 8 mm of diameter on either side along the back midline of the ICR mice was adopted. The S_4_Q_4_G_4_ hydrogel with the best adhesion effect and flexibility for the skin was chosen as a representative, and a commercial Tegaderm® dressing was used as a control group. The representative photographs of the healing process and traces of wound-bed closure during 21 days are shown in Figures 5A,B. Obviously, the wound areas in both groups became smaller with increasing post-surgery time, but the hydrogel group showed significantly faster contraction than that of the control group from day 7 onward. Moreover, no erythema or edema formation was observed on the wounds over the whole repair process, further suggesting the excellent antibacterial effect and biocompatibility of the SQG hydrogel as a wound dressing. This was also supported by the skin irritation results (Supplementary Figure S5). Quantitative analysis of the wound area throughout the treatment period is shown in Figure 5C. The wounds covered with the S_4_Q_4_G_4_ hydrogel significantly decreased to 51.9% and 16.4% compared with 83.4% and 64.3% of the control group on day 7 and day 14, respectively. The average wound healing time for the hydrogel group was 16 days, while the control group needed 24 days at least (Figure 5D, *p < 0.5). These results indicate that the S_4_Q_4_G_4_ hydrogel could accelerate the healing kinetics, thereby improving tissue regeneration.

To evaluate the S_4_Q_4_G_4_ hydrogels for granulation tissue, histological samples were taken at days 7, 14, and 21 post-wounding and examined via H&E, Masson’s trichrome stains, and Picrosirius red staining, followed by a quantitative analysis of the histological sections (Figure 5E, Supplementary Figure S6). H&E staining results show that the entire defect region treated by hydrogels has almost been filled by granulation tissue after 7 days of surgery compared with the control group. On day 14, a full dermis layer and a nearly closed epithelial layer formed in the wound area treated with hydrogel, while a bulk of incrustation was clearly observed in the control group. The statistical results showed that the thickness of granulation tissue in the hydrogel group was significantly higher than that in the control group on the 7th and 14th days (Supplementary Figure S6A). Moreover, abundant new blood vessels (indicated by black arrows) have been generated on the wound sites in the hydrogel group, indicating that the SQG hydrogel is beneficial to promote angiogenesis (Supplementary Figure S6B). After the treatment of 21 days, new regenerative skin perfectly connected with the normal tissues in the hydrogel group, whereas the wound still was not fully closed in the control group. Masson’s trichrome staining shows that the granulation tissues in the hydrogel group always contained a larger amount of collagen fibers in comparison with those in the control group. These collagen fibers were further assessed by Picrosirius red staining and observed under a polarized light microscope, where type I collagen was identified as thick orange/yellow fibers and type III collagen as thin greenish fibers. Wounds treated with hydrogel show a higher ratio of collagen type III/I, intrinsically indicating a scarless tissue regeneration. On days 7, 14, and 21, the hydrogel group continually exhibited a faster collagen deposition than that of the control group (Supplementary Figure S6C). This indicates that the SQG hydrogels remarkably accelerate wound closure. In summary, treatment of wounds with the SQG hydrogels exhibited an enhanced granulation tissue generation and ECM remodeling.

## Conclusion

In summary, we demonstrate a novel skin-adhesive zwitterionic poly(sulfobetaine methacrylate) (PolySBMA) hydrogel cross-linked by QCSMA and GelMA for wound repair. The proposed hydrogel showed high flexibility with tensile strength and break strain as high as 310.5 kPa and 360%, respectively. Chemical bonding and amino protonation of QCSMA in the network synergistically account for the low swelling ratio (313.7%) of the hydrogel. Moreover, abundant ion–dipole and dipole–dipole interactions in the network provided a universal adhesion of hydrogel with a variety of substrates. The optimal adhesion strength of hydrogel adhered on the skin surface is up to 38.2 kPa. With the assistance of PolySBMA and QCSMA, the hydrogel exhibited a broad-spectrum antibacterial property against Gram-positive and Gram-negative bacteria. Either *in vitro* or *in vivo* results demonstrated that the hydrogel has high cellular activity, good hemocompatibility, and histocompatibility. These excellent overall performances are beneficial for the hydrogel as wound dressing for cutaneous defect repair. Compared with the commercial Tegaderm film, the hydrogel exhibited a better healing effect in terms of wound closure, neovascularization, granulation tissue, and collagen deposition. In conclusion, such a flexible, skin-adhesive antibacterial hydrogel holds great promise for the treatment of skin wounds.

## Data Availability

The raw data supporting the conclusions of this article will be made available by the authors, without undue reservation.
