# Supplementary material for: Highly Adhesive Antibacterial Bioactive Composite Hydrogels With Controllable Flexibility and Swelling as Wound Dressing for Full-Thickness Skin Healing
Source: Front Bioeng Biotechnol. 2021 Dec 23;9:785302. doi: 10.3389/fbioe.2021.785302 (PMC8735859; doi:10.3389/fbioe.2021.785302)
Supplement: Supplementary file 7 [file DataSheet1.docx]

Supplementary Material

Highly Adhesive Antibacterial Bioactive Composite Hydrogels with Controllable Flexibility and Swelling as Wound Dressing for Full-Thickness Skin Healing

Guanhua Lan^1^, Suping Zhu^2^, Dong Chen^3^, Hua Zhang^4*^, Lijin Zou^1*^, Yuanlin Zeng^1*^

^1^Burn Center, The First Affiliated Hospital of Nanchang University, Nanchang, Jiangxi, 330006, China

^2^Department of pediatrics, Ningbo Yinzhou Second Hospital, Ningbo, Zhejiang, 315100, China

^3^Department of pathology, Ningbo Yinzhou Second Hospital, Ningbo, Zhejiang, 315100, China

^4^Cixi Institute of Biomedical Engineering, Ningbo Institute of Materials Technology and Engineering, Chinese Academy of Sciences, Ningbo 315300, China

*** Correspondence:**Yuanlin Zeng
[zengyl777@126.com@uni.edu](mailto:zengyl777@126.com@uni.edu)

Lijin Zou
[zou.li.jin@hotmail.com](mailto:zou.li.jin@hotmail.com)

Hua Zhang
[zhanghua@nimte.ac.cn](mailto:zhanghua@nimte.ac.cn)


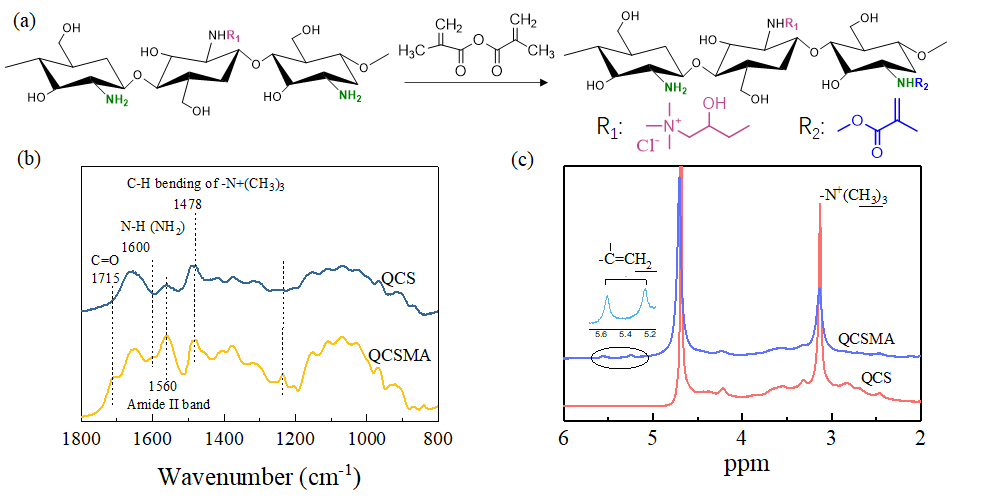


**Supplementary Figure 1.** Synthesis and structure characterization of water-soluble quaternized chitosan (QCS) and quaternized chitosan methacrylate (QCSMA). (a) Schematic illustration to the N-acylation reaction between QCS and methacrylic anhydride. (b) FTIR spectra of QCS and QCSMA. (c) ^1^H NMR spectra of QCS and QCSMA dissolved in D_2_O.


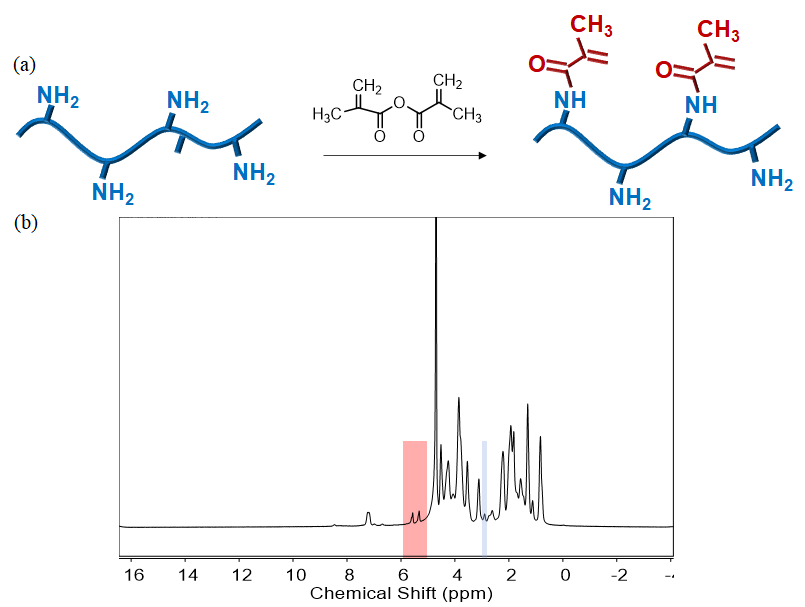


**Supplementary Figure 2. (a)**Schematic illustration to the N-acylation reaction between gelatin and methacrylic anhydride. (b) ^1^H NMR spectra of gelatin methacrylate (GelMA) dissolved in D_2_O.


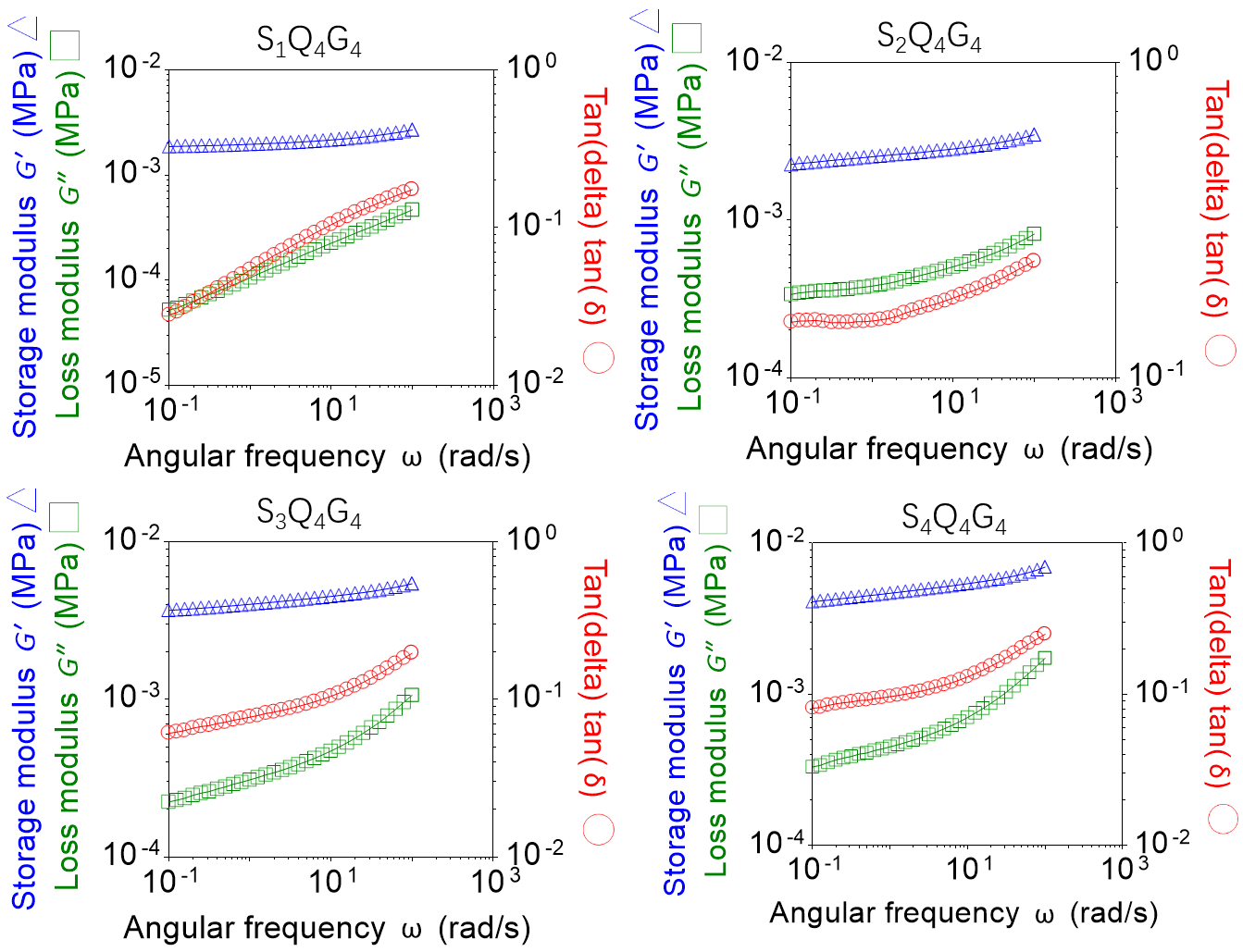


**Supplementary Figure 3.** Frequency dependency of storage modulus (G’) and loss modulus (G’’) of S_1_Q_4_G_4_, S_2_Q_4_G_4_, S_3_Q_4_G_4_, and S_4_Q_4_G_4_ hydrogels.

**
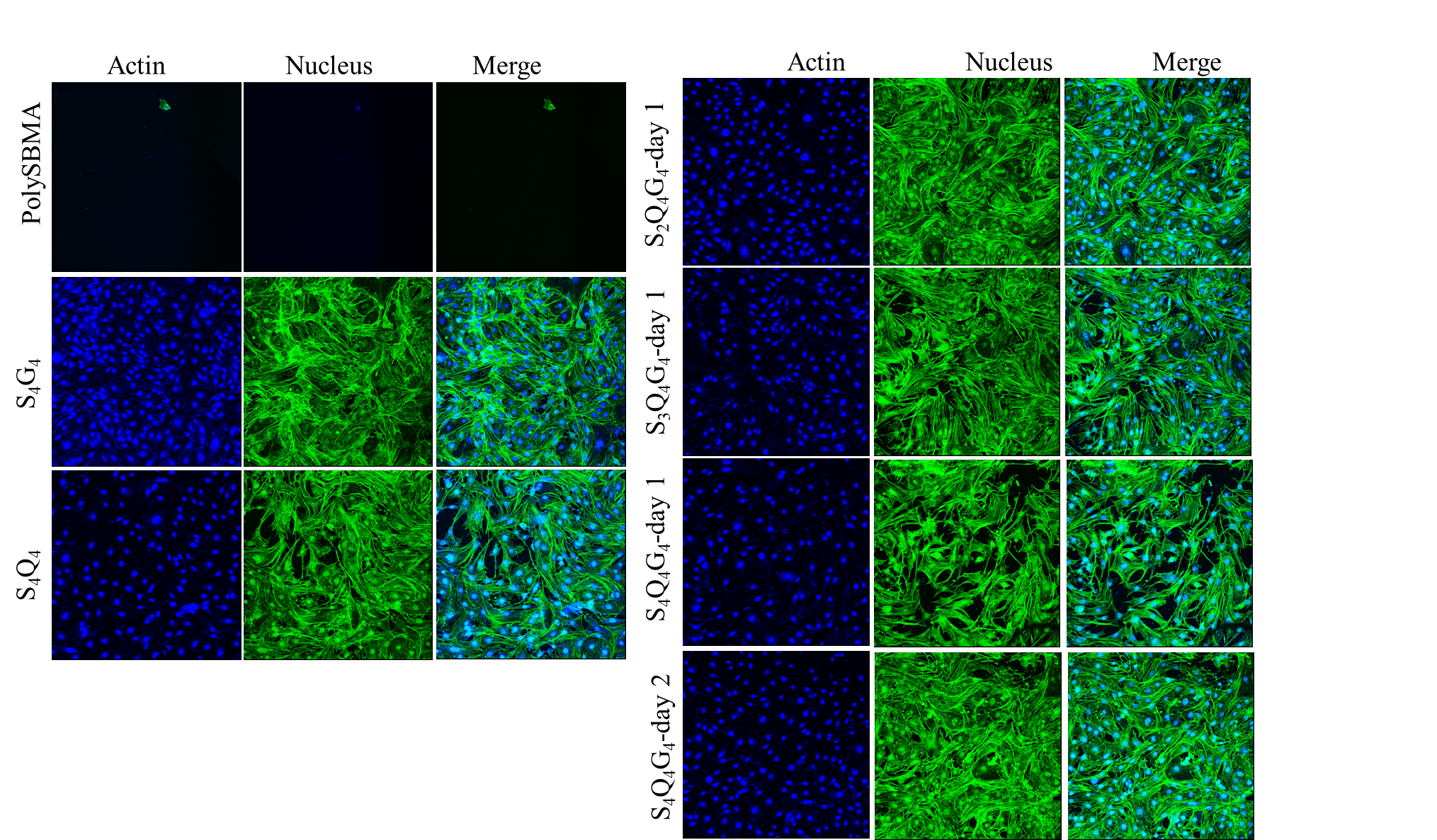
**

**Supplementary Figure 4.** Fluorescent staining (Phalloidin-FITC for actin, DAPI for nucleus) of 3T3 cells seeded on the PolySBMA, S_1_G_4_, S_1_Q_4_, S_1_Q_4_G_4_, S_2_Q_4_G_4_, S_3_Q_4_G_4_, and S_4_Q_4_G

**Videos**

**Video S1.** The SQG hydrogels exhibited enhanced tensile performances compared to the PolySBMA hydrogels only with QCSMA or GelMA chains

**Video S2.** The effect of the zwitterionic monomer content on adhesion performance through hanging a load of 1.0 kg by the hydrogel

**Video S3.** Adhesion properties of the S_4_Q_4_G_4_ hydrogel to a load of 1.0 kg in water.

**Video S4.** Adhesion properties of the S_4_Q_4_G_4_ hydrogel and wet gauze on the back of an alive ICR mouse

**Video S5.** Adhesion properties of the S_4_Q_4_G_4_ hydrogel on the hand of a volunteer rotating the arm
